# Anti-Microbial, Anti-Biofilm Activities and Cell Selectivity of the NRC-16 Peptide Derived from Witch Flounder, *Glyptocephalus cynoglossus*

**DOI:** 10.3390/md11061836

**Published:** 2013-05-28

**Authors:** Ramamourthy Gopal, Jun Ho Lee, Young Gwon Kim, Myeong-Sun Kim, Chang Ho Seo, Yoonkyung Park

**Affiliations:** 1 Research Center for Proteineous Materials, Chosun University, Gwangju 501-759, Korea; E-Mail: ramagopa@gmail.com; 2 Department of Biotechnology, Chosun University, Gwangju 501-759, Korea; E-Mails: juno6267@hanmail.net (J.H.L.); kyg1022@hanmail.net (Y.G.K.); kimsun59@nate.com (M.-S.K.); 3 Department of Bioinformatics, Kongju National University, Kongju 314-701, Korea; E-Mail: chseo@kongju.ac.kr

**Keywords:** fish peptide, NRC-16, antimicrobial peptide, antibiofilm peptide, eukaryotic membrane, phosphatidylcholine, cholesterol, sphingomyelin

## Abstract

Previous studies had identified novel antimicrobial peptides derived from witch flounder. In this work, we extended the search for the activity of peptide that showed antibacterial activity on clinically isolated bacterial cells and bacterial biofilm. *Pseudomonas aeruginosa* was obtained from otitis media and cholelithiasis patients, while *Staphylococcus aureus* was isolated from otitis media patients. We found that synthetic peptide NRC-16 displays antimicrobial activity and is not sensitive to salt during its bactericidal activity. Interestingly, this peptide also led to significant inhibition of biofilm formation at a concentration of 4–16 μM. NRC-16 peptide is able to block biofilm formation at concentrations just above its minimum inhibitory concentration while conventional antibiotics did not inhibit the biofilm formation except ciprofloxacin and piperacillin. It did not cause significant lysis of human RBC, and is not cytotoxic to HaCaT cells and RAW264.7 cells, thereby indicating its selective antimicrobial activity. In addition, the peptide’s binding and permeation activities were assessed by tryptophan fluorescence, calcein leakage and circular dichroism using model mammalian membranes composed of phosphatidylcholine (PC), PC/cholesterol (CH) and PC/sphingomyelin (SM). These experiments confirmed that NRC-16 does not interact with any of the liposomes but the control peptide melittin did. Taken together, we found that NRC-16 has potent antimicrobial and antibiofilm activities with less cytotoxicity, and thus can be considered for treatment of microbial infection in the future.

## 1. Introduction

*Pseudomonas aeruginosa* and *Staphylococcus aureus* strains are known to be opportunistic pathogens that cause some of the most prevalent infections in eye, ear, wound and lung [1]. These pathogens are endowed with a wide range of drug resistance properties [2,3,4,5] and are capable of forming a biofilm matrix, which acts as a barrier for bacterial cells against antibiotics, host immune cells and antimicrobial factors [6,7,8,9]. However, the cationic antimicrobial peptides (AMPs) represent a new class of antibiotics, because they are entirely different from the antibiotics that eliminate pathogens. While antibiotics have definite intracellular targets for their activity, AMPs generally do not have a specific target in the microbial cell [10,11]. Instead, they bind to the bacterial cell membrane and perturb the membrane structure. Indeed, some AMPs show selective inhibition of intracellular targets inside the microbial cells [12]. This action renders AMPs impregnable to bacterial resistance, for which the microbes need to change the entire membrane lipid composition. Therefore, AMPs are attractive for their potential therapeutic effect against drug-resistant organisms.

Marine peptides have been shown to possess antimicrobial, antiviral, anticoagulant and antifreeze properties by recent research, and the number of AMPs isolated from marine organisms is growing. These AMPs are found in a range of phyla including Mollusca, Crustacea, Porifera, Cnidaria as well as a number of fish species [13,14,15,16,17]. However, marine fish is one of the richest sources of this type of peptide. Although researchers evaluated the activities of different AMPs from fish, their data suggest that pleurocidin, piscidins and pardaxin peptides can serve as attractive molecules for the development of new therapeutic strategies to fight life-threatening infectious diseases [18]. Therefore, we focused on pleurocidin-like cationic AMP, NRC-16 (GWKKWLRKGAKHLGQAAIK-NH_2_), a peptide truncated from NRC-17 (GWKKWLRKGAKHLGQAAIKGLAS), which is identified from the witch flounder [19]. The witch flounder, *Glyptocephalus cynoglossus*, is a right-eyed flatfish of the family Pleuronectidae.

**Figure 1:**
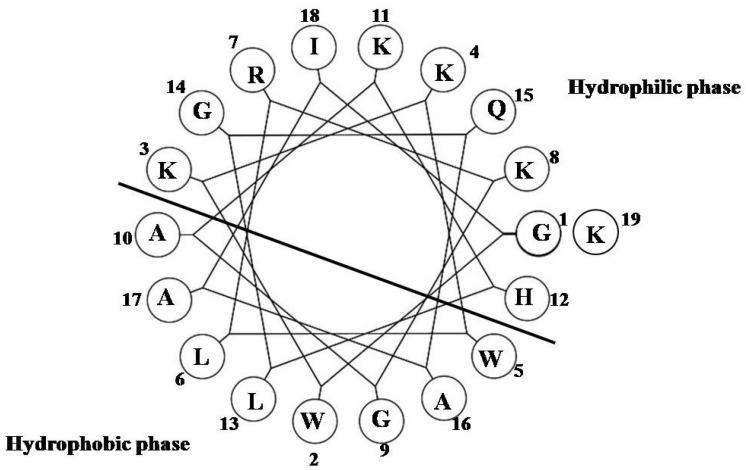
Helical wheel diagram of NRC-16.

NRC-16 has the following amino acid sequence: Four polar uncharged amino acids (three glycines (Gly) and one glutamine (Gln)), seven polar charged residues (five lysines (Lys), one arginine (Arg) and one histidine (His)) and eight hydrophobic residues (two tryptophans (Trp), three alanines (Ala), two leucines (Leu) and one isoleucine (Ile)). This structure has enough potential to provide the greatest degree of amphipathicity (Figure 1) or hydrophobicity and largest cationicity. NRC-16 also has amidated *C*-termini that greatly improve microbicidal activity of the peptide [20]. In previous studies, NRC-16 was shown to exert a potent growth inhibitory effect against Gram-negative bacteria, Gram-positive bacteria and fungi [19,21]. Given this property of NRC-16, the aim of the present study is to establish its activity against multidrug-resistant (MDR) bacteria like *P. aeruginosa* obtained from patients in whom otitis media and biofilm inhibition were observed. We synthesized NRC-16 peptide that showed antimicrobial activity and inhibited biofilm formation with no cytotoxic effects.

## 2. Results and Discussion

### 2.1. Lytic Effects of NRC-16

Development of new types of antibiotic compounds is an exciting area of research. Numerous studies have demonstrated that AMPs can be the next line of compounds to overcome bacterial resistance [12,22]. AMPs are now one of the most promising candidates against MDR bacterial strains. For these reasons, we assessed the antimicrobial activity of NRC-16 using 96-well plate as an indication of *in vitro* assays that were used to measure the antimicrobial activity of NRC-16 against three strains of Gram-negative bacteria, two strains of Gram-positive bacteria and fungal cells, and 32 strains of antibiotic-resistant bacteria including *P. aeruginosa* and *S. aureus*. We also tested the effects of one of the AMPs, melittin, which acted as a control peptide. As shown in Table 1, the minimum inhibitory concentrations (MICs) of NRC-16 ranged from 2 to 16 μM in low (sodium phosphate (SP) buffer) and high ionic strength buffer (phosphate-buffered saline (PBS)) against nearly all standard strains of bacteria and fungal cells. The antimicrobial activity of the two peptides was slightly inhibited in the presence of PBS (Table 1). NRC-16 also exerts potent antimicrobial activity against a wide variety of drug-resistant *E. coli*, *S. typhimurium* and *S. aureus* (Table 1). The MDR *P. aeruginosa* and *S. aureus* strains were of critical concern among the strains tested (Table 2). The results indicated that the peptide was very effective against both *P. aeruginosa* and *S. aureus* strains. The order of activity of NRC-16 is similar to that of fish peptides such as pleurocidin and piscidins, showing a good activity against MDR, and thus opening up the possibility of identification and isolation of other peptides from fish or marine organisms [23,24]. We show here that NRC-16 notably exerted both antibacterial and antifungal activity against antibiotic-resistant strains like piscidin and pleurocidin [24], which may decrease the chance of candidal superinfections normally associated with bacterial infection on skin such as in atopic dermatitis [25].

Antimicrobial assays were performed in 10 mM SP buffer, pH 7.2 and PBS, pH 7.2 (number in parentheses in the Table 1 represents MICs of NRC-16 in PBS against standard strains of bacteria and fungal cells). 

**Table 1:** Antimicrobial activity of NRC-16.

MIC (µM)		
Microorganism	NRC-16	Melittin
**Gram (−) bacteria**		
*E. coli*	2(4)	2(4)
*S. typhimurium*	1(2)	2(2)
*P. aeruginosa*	4(8)	8(16)
**Gram (+) bacteria**		
*S. aureus*	4(8)	2(2)
*B. subtilis*	2(8)	1(1)
Yeast		
*C. albicans*	8(16)	8(16)
*T. beigelli*	4(8)	2(4)
**Resistant strains ^a^**		
*E. coli* CCARM 1229 ^b^	8	2
*E. coli* CCARM 1238 ^b^	4	2
*S. typhimurium* CCARM 8007 ^c^	4	8
*S. typhimurium* CCARM 8009 ^c^	16	16
*S. typhimurium* CCARM 8013 ^c^	4	8
*S. aureus* CCARM 3089 ^d^	2	2
*S. aureus* CCARM 3090 ^d^	8	8
*S. aureus* CCARM 3108 ^d^	2	2
*S. aureus* CCARM 3114 ^d^	4	2
*S. aureus* CCARM 3126 ^d^	4	8
*C. albicans* CCARM 14001 ^e^	8	4

^a^ Resistant strains (except *C. albicans*) were performed according to the National Committee for Clinical Laboratory Standards (NCCLS) method [26,27]; ^b^ *E. coli* 1229 and 1238; ^c^ *S. typhimurium* 8007, 8009 and 8013 are resistant-strains to ampicillin; ^d^ *S. aureus* 3089, 3090, 3108, 3114 and 3126 are resistant to oxacillin. ^e^ *C. albicans* 14001 are resistant to fluconazol.

To determine the bactericidal action of NRC-16 in the presence of salt, NaCl is added in Mueller Hinton broth (MHB) up to a concentration of 200 mM. NaCl did not affect the bactericidal activity of NRC-16 against *P. aeruginosa* 1034 and 4007 (data not shown), indicating that NaCl ions were not inhibiting the peptide’s bactericidal action, unlike salt-sensitive activities of several peptides including beta defensin [28,29], magainin [30] and LL-37 [31]. However, not all AMPs are salt sensitive, and some peptides show potent salt-insensitive antimicrobial activities (e.g., clavanin, and tachyplesins) [30,32]. NRC-16’s action is also consistent with other fish peptide pleurocidin, which is not sensitive to salt conditions such as NaCl, MgCl_2_ and CaCl_2_ [23]. 

**Table 2:** Antimicrobial activity of NRC-16 against clinically isolated strains.

MIC (µM)		
Resistant strains	NRC-16	Melittin
*P. aeruginosa* 1034 ^a^	4	4
*P. aeruginosa* 1162 ^a^	2	2
*P. aeruginosa* 3399 ^a^	2	2
*P. aeruginosa* 3547 ^a^	4	8
*P. aeruginosa* 3592 ^a^	8	8
*P. aeruginosa* 4007 ^a^	2	2
*P. aeruginosa* 4076 ^a^	8	8
*P. aeruginosa* 5018 ^a^	4	8
FRPA ^b^	8	16
CRPSP ^c^	8	16
IRPA ^d^	4	16
*S. aureus* 254348 ^e^	2	2
*S. aureus* 254422 ^e^	1	1
*S. aureus* 691054 ^e^	2	4
*S. aureus* 949987 ^e^	2	2
*S. aureus* 950805 ^e^	1	8
*S. aureus* 2-660 ^e^	8	2
*S. aureus* 3518 ^e^	8	4
*S. aureus* 2-3566 ^e^	4	4
*S. aureus* 2-777 ^e^	4	2
*S. aureus* 2-3122 ^e^	4	2
*S. aureus* 2-254 ^e^	4	2

^a^ *P. aeruginosa* are resistant strains isolated from patients with otitis media in a hospital; ^b^ FRPA: Flomoxef sodium-resistant *P. aeruginosa*; ^c^ CRPSP: Cefrpiramide-resistant *P. aeruginosa*; ^d^ IRPA: Isepamicin-resistant *P. aeruginosa*; ^e^ *S. aureus* are resistant strains isolated from patients in a hospital.

### 2.2. Effect of AMPs on the *P. aeruginosa* Biofilm

In addition to ear infections, and lung infections in cystic fibrosis patients, caused by *P. aeruginosa* [9,33], it is an environmental organism that regularly causes both acute and chronic infections and is one of the leading causes of morbidity and mortality in thermally injured patients [34,35,36]. Moreover, *P. aeruginosa* is capable of causing biofilm infection in a wound [37,38]. It is likely that a wound environment is able to support the development of bacterial biofilms because when thermal injury damages the skin, which paves the way for bacteria, the host’s immune system becomes suppressed. Within the wound, MDR bacteria often develop biofilms that could have a significant effect on inflammation, infection and healing [39]. Interestingly, it has been shown that *P. aeruginosa* formed a biofilm in the thermal mouse model of acute infections [40]. We cultured the *P. aeruginosa* in 96 wells, allowing the formation of biofilm by the production of an extracellular matrix of various polysaccharides, macromolecules and the up-regulation of a number of genes involved in surface attachment [41,42]. We then applied antimicrobial agents to evaluate their inhibitory effect on the biofilm growth. This study revealed that *P. aeruginosa* strains isolated from otitis media were highly resistant to important antibiotics (ampicillin, chlorophenical, erythromycin, levofloxacin and ciprofloxacin) (Table 3), as no inhibition was observed. The resistance of MDR *P. aeruginosa* strains to ciprofloxacin is consistent with the previous report [43]. However, pipracillin at a higher concentration showed biofilm inhibition activity, which, in agreement with previous studies results, indicated that high concentrations of antibiotics are needed to drive the antibiofilm activity [44,45]. Bacteria forming biofilms may be up to 1000 times more resistant to antimicrobial agents than those in a planktonic state [46]. Remarkably, NRC-16 and melittin peptides showed elevated inhibitory effect on all the strains of *P. aeruginosa* isolated from patients with otitis media (Table 2), with a minimum biofilm inhibition concentration (MBIC) in the range of 4–16 μM (Table 3). The various AMPs such as LL-37, MUC7, G10KHc, colistin, truncated LL-37 and pleurocidin showed antibiofilm activity [47,48,49,50,51,52]. The antimicrobial and antibiofilm actions of NRC-16 merit further study. Thus far, application of AMPs in the clinical setting has been hampered due to various reasons, and most therapeutic peptides are being developed for topical uses only, with the exception of the anionic lipodepsipeptide daptomycin [53]. Moreover, research on AMPs from fish generally focuses on the development of topical application for dermatological disease [18], and we also showed that NRC-16 peptide interacted with gelatin, which can have a wound-healing application [21]. These results imply that the presence of peptide in the gelatin matrix may inhibit *P. aeruginosa* biofilm in a wound environment. Other studies also described that topical antimicrobial protection applied in the biofilm colonized the wound surface [54,55,56].

**Table 3:** Inhibitory effects of NRC-16, melittin and antibiotics on the biofilm strains of *P. aeruginosa*.

MBIC (μM)								
Strains	Amp	Chl	Ery	Lev	Cip	Pip	NRC-16	Melittin
**1162**	>512	>512	>512	>512	256	128	8	4
**3547**	>512	>512	>512	>512	512	256	8	16
**4007**	>512	>512	>512	>512	512	128	16	4
**3399**	>512	>512	>512	>512	>512	256	8	4
**1034**	>512	>512	>512	>512	>512	128	16	8

Amp, Chl, Ery, Lev, Cip and Pip are ampicillin, chloramphenicol, levofloxacin, ciprofloxacin and piperacillin, respectively.

### 2.3. Hemolytic and Cytotoxicity Activity of Peptides

**Figure 2:**
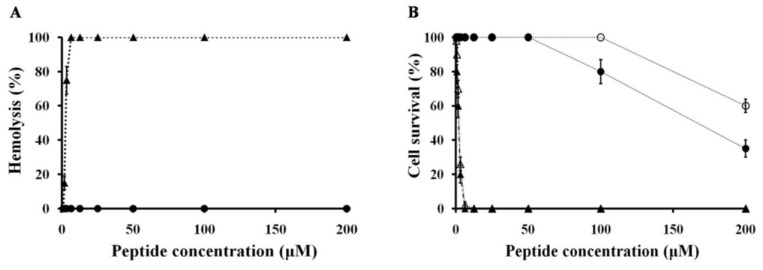
Cytotoxicity towards hRBCs, HaCaT cells and Raw264.7 cells. (**A**) Dose-dependent release of hemoglobin measured after incubating hRBCs (final RBC concentration, 4% v/v) for 1 h with NRC-16 (circles) or melittin (triangles); (**B**) HaCaT cells (filled) or Raw264.7 cells (4 × 10^3^ cell/well) were incubated for 24 h with the indicated concentration of NRC-16 (circles) or melittin (triangles), after which percent cell survival was determined in MTT assays. All graphs show mean values obtained from at least three independent experiments performed in duplicate.

In the present study, NRC-16 peptide exhibited good activity against antibiotic-resistant bacterial strains, including biofilm cells. However, their cytotoxicity against mammalian cells (human red blood cells (hRBCs), HaCaT and RAW cell 264.7) should be tested. An effective peptide that can be used for human skin disease treatment should be non-cytotoxic and non-hemolytic [57]. The cytotoxicities of NRC-16 and melittin were tested (Figure 2), against hRBCs (human red blood cells), HaCaT cells (human skin keratinocytes cells) and RAW264.7 cells (morphologically monocytes and macrophages). The hemolytic activities of the NRC-16 and melittin are entirely different because the NRC-16 is inactive until 150 μM, whereas melittin showed highest hemolytic activity even at 10 μM. Similarly, NRC-16 was not cytotoxic towards HaCaT cells and RAW264.7 cells up to 50 μM, while melittin showed cytotoxicity even at 5 μM. This clearly indicated that NRC-16, unlike melittin, has antimicrobial activity without a high degree of hemolysis. This may reflect an optimal balance of cationicity and hydrophobicity, which are features needed for antimicrobial activity without hemolysis [58,59].

### 2.4. NRC-16 is Non-Selective against Eukaryotic Membranes Using Liposomes

The difference in activity of melittin and NRC-16 on mammalian cells may lie in their difference in membrane affinity. Therefore, we investigated the effects of these peptides on model vesicles composed of zwitterionic phospholipids, including phosphatidylcholine (PC), PC:cholesterol (CH) (2:1, w/w) and PC:sphingomyelin (SM) (2:1, w/w) as these three lipids are the major constituents of most mammalian membranes. We used different methods such as characterization of Trp environment using fluorescence spectroscopy, calcein leakage and circular dichroism (CD). 

**Figure 3:**
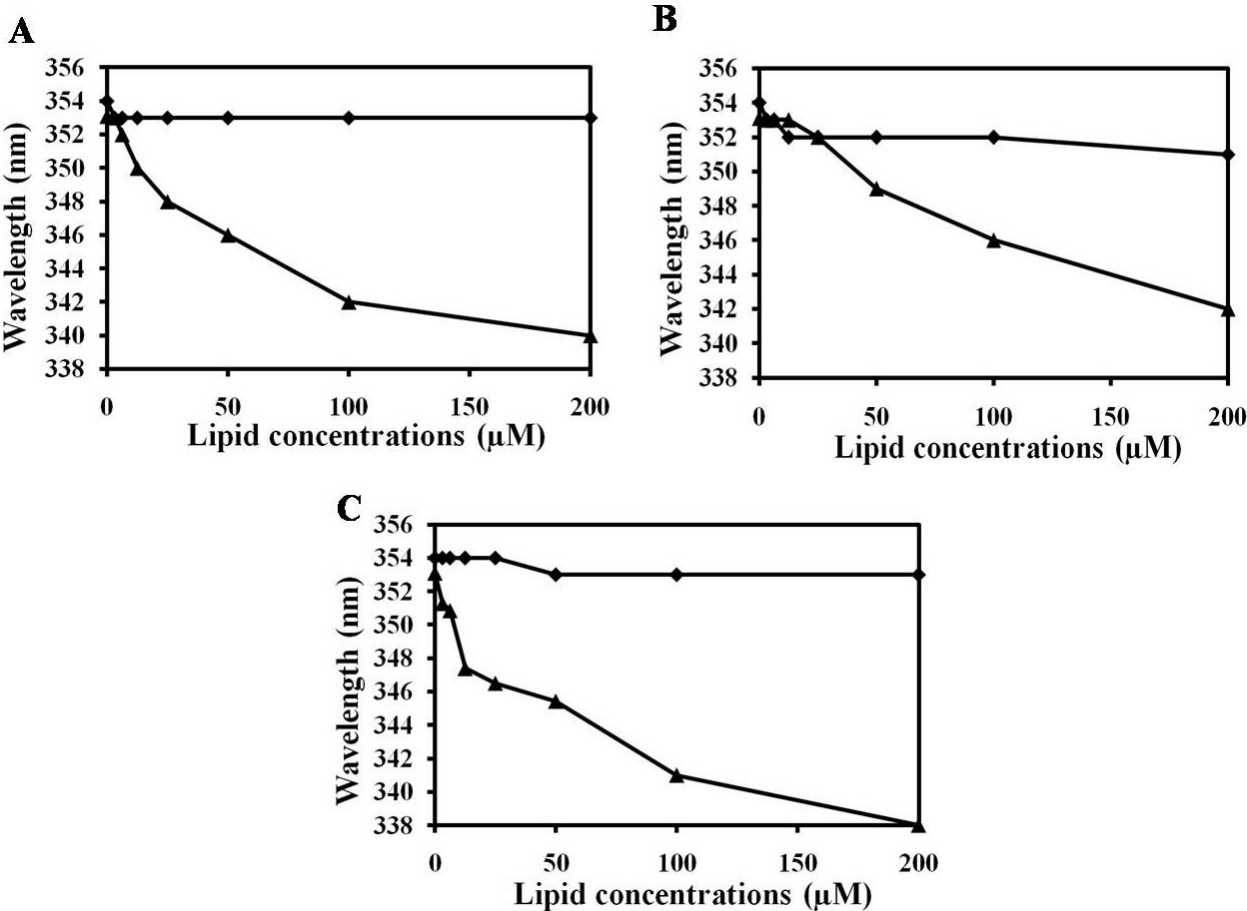
Blue shift in Trp fluorescence. Emission maxima from Trp in peptides (2 μM) in the presence of (**A**) 200 μM PC; (**B**) 200 μM PC:CH (2:1, w/w); and (**C**) 200 μM PC:SM (2:1, w/w). NRC-16 and melittin are represented by squares and triangles, respectively. All experiments were conducted at 25 °C.

**Figure 4:**
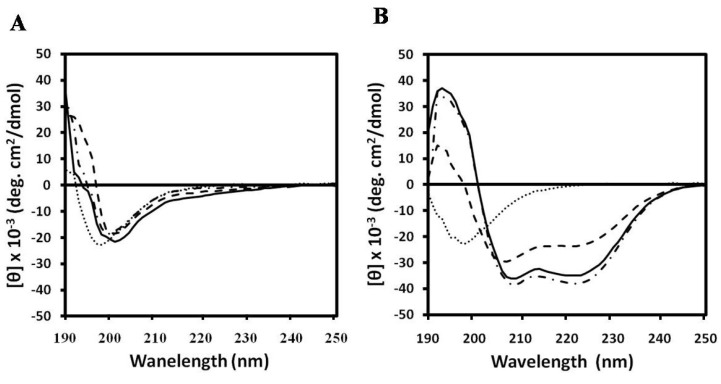
CD spectra for the peptides (50 μM) were measured in the presence of PBS (pH 7.2), 1 mM PC, 1 mM PC:CH (2:1, w/w) and 1 mM PC:SM (2:1, w/w). CD spectra of NRC-16 (**A**) and melittin (**B**) in aqueous solution (dotted line) as well as in the presence of PC (solid line), PC:CH (2:1, w/w) (dashed line) and PC:SM (2:1, w/w) (dashed-dotted line).

To evaluate whether Trp residues have a preference for the interfacial region of lipid bilayers [60,61,62], the Trp fluorescence emission can be followed. When Trp residues move from an aqueous environment (polar) to a membrane environment (less polar) the Trp fluorescence emission spectra has a blue shift. Therefore, the peptide fluorescence emission spectra were followed with increasing concentrations of lipid membrane to evaluate if the Trp is inserting in the lipid membrane. The Trp fluorescence blue shift assay indicated that NRC-16 peptide did not bind with PC, PC:CH (2:1, w/w) and PC:SM (2:1, w/w) (Figure 3). The calcein leakage (data not shown) and CD data (Figure 4) also indicated that NRC-16 had no interaction with all liposomes, which is consistent with its lack of hemolytic activity in erythrocytes [63,64,65,66]. In fact, NRC-16 peptide interacts with negatively charged sodium dodecyl sulfate (SDS) through electrostatic interaction initially experiences accumulation on the bilayer surface [21]. Moreover, NRC-16 peptide adopted an alpha-helical structure in the lipopolysaccharides (LPS) [21]. This implies that NRC-16 interact with the negatively charged surface molecules, such as LPS of Gram-negative bacteria and SDS. However, NRC-16 did not strongly bind with any of the PC, CH and SM liposomes because of its optimum balance of charge/hydrophobicity; a property that prevented the peptide from undergoing electrostatic/hydrophobic interactions with zwitterionic membranes, resulting in low toxicity towards mammalian cells (Figure 2). In contrast, melittin showed a strong interaction with all liposomes. Indeed, melittin adopted appreciable secondary structure in the presence of a mammalian mimetic membrane and caused membrane disruption, which could be associated with its cytotoxic activity [67]. 

## 3. Experimental Section

### 3.1. Materials

Rink amide 4-methylbenzhydrylamine resin, fluoren-9-ylmethoxycarbonyl (Fmoc) amino acids, and other reagents for peptide synthesis were purchased from Calibochem-Novabiochem (La Jolla, CA, USA). CH from porcine liver, egg yolk l-α-PC and SM were obtained from Avanti Polar Lipids, Co. (Alabaster, AL, USA). Calcein was obtained from Molecular Probes (Eugene, OR, USA). All other reagents were of analytical grade. All buffers were prepared using double distilled water (Millipore Co., Bedford, MA, USA).

### 3.2. Peptides Synthesis

Peptides were synthesized and purified as reported previously [21,68]. The purity of these peptides was found to be >95%. The concentrations of the peptides were calculated by UV absorption at 280 nm using extinction coefficients determined by ProtParam [69].

### 3.3. Antibacterial Activity

The antibacterial activity of the peptides against Gram-negative and Gram-positive strains was examined using the microbroth dilution method [68]. Aliquots of bacterial suspensions in mid-logarithmic phase at a concentration of 2 × 10^5^ colony forming units (CFU)/mL in culture medium were added to each well, containing peptide solutions diluted with two-fold serial in 10 mM SP buffer (pH 7.2) or PBS (1.5 mM KH_2_PO_4_, 2.7 mM KCl, 8.1 mM Na_2_HPO_4_, 150 mM NaCl, pH 7.2). Inhibition of growth was determined by measuring absorbance at 620 nm using a Versa-Max microplate Elisa Reader (Molecular Devices Co., Sunnyvale, CA, USA) after incubation for 24 h at 37 °C. The MIC is defined as the minimal peptide concentration that completely inhibits bacterial growth. *E. coli* (KCTC 1682), *S. typhimurium* (KCTC 1926), *P. aeruginosa* (KCTC 1637), *S. aureus* (KCTC 1621), and *B. subtilis* (KCTC 1918) were obtained from the Korean Collection for Type Cultures (KCTC, Daejeon, Korea).

### 3.4. Antifungal Activity

To assess the activity of NRC-16 against various fungal pathogens, fungal spores (*C. albicans* (KCTC 7270) and CCARM (Culture Collection of Antibiotic-Resistant Microbes) 14001 and *T. beigelli* (KCTC 7707)) from seven-day-old cultures grown on potato dextrose broth (PDA) plates at 25 °C were collected using 0.08% Triton X-100 [70]. Its surfactant property disperses the spore clumps from the PDA media [71]. Yeast cultures grown overnight were then suspended in PDA media. Spore concentrations were then adjusted to 5 × 10^4^ spores/mL in medium composed of half PDA media and half appropriate buffer, after which 80 μL was added to the wells of sterile 96-well flat-bottomed microtiter plates along with 20 μL of peptide or media to give final concentrations of 1–64 μM/mL. After incubating for 24–36 h at 25 °C, the lowest concentration of peptide-inhibiting fungal growth was microscopically determined as the MIC [72]. 

### 3.5. *In Vitro* Activity of the NRC-16 Peptide against Clinical Isolated *P. aeruginosa* and *S. aureus* Strains

The antimicrobial activity of each peptide was tested using a broth microdilution assay following the procedure recommended by the NCCLS, with slight modifications [31,32]. Briefly, bacteria were grown to the stationary phase overnight in MHB at 200 rpm and 37 °C. The cultures were then diluted with fresh MHB to a final concentration of 2 × 10^5^ CFU/mL. Next, a 256 μM/mL stock solution of each peptide was prepared in 0.01% acetic acid and 0.2% bovine serum albumin (BSA) in a polypropylene microtube. The peptide solution was then subjected to a series of two-fold dilutions in 0.01% acetic acid and 0.2% BSA until reaching a final concentration of 1–64 μM/mL. Next, 100-μL aliquots of the microbial suspension were dispensed into each well of a 96-well polypropylene microtiter plate (Costar 3790; Corning, NY, USA), after which 10 μL of the peptide solutions were added. After 24 h of incubation at 37 °C, the antibacterial activities of the peptides were assessed based on optical densities (ODs) in each well. Absorbance of the optical densities was recorded at 620 nm using a Versa-Max microplate Elisa Reader (Molecular Devices Co., Sunnyvale, CA, USA). The MIC was expressed as the lowest concentration that inhibited the cell growth. There were three replicates for each test sample. The MBC was determined by streaking a 5 μL aliquot of the microtiter plate reaction mixture onto an MHB agar plate for the three serial dilution wells above and below the determined MIC. The lowest concentration of NRC-16 that abated the bacterial colony growth on the agar plate was determined the MBC [23]. Drug-resistant *E. coli* strains (CCARM 1229 and CCARM 1238), *S. typhimurium* strains (CCARM 8007, CCARM 8009 and CCARM 8013) and *S. aureus* strains (CCARM 3089, CCARM 3090, CCARM 3108, CCARM 3114 and CCARM 3126) were obtained from the CCARM at Seoul Women’s University in Korea. A total of three clinical isolates of *P. aeruginosa* strains that are resistant to flomoxef sodium, isepamicin and cefpiramide were collected from the Hanyang University Hospital, in Guri-city, South Korea. *P. aeruginosa* 1034, 1162, 3399, 3547, 3592, 4007, 4076 and 5018 were resistant strains isolated from patients with otitis media in a hospital. *S. aureus* 254348, 254422, 691054, 949987, 950805, 2-660, 3518, 2-3566, 2-777, 2-3122 and 2-254 were resistant strains isolated from patients in a hospital. All isolates were stored at −70 °C until required.

### 3.6. Biofilm Forming Strains Subjected to Susceptibility Assay with NRC-16, Melittin and Some Conventional Antibiotics

To examine the inhibitory effect of test agents (NRC-16, melittin and antibiotics) on the biofilm growth, the tissue culture plate (TCP) method was employed with a few modifications [73]. Bacterial strains were cultured in a MHB supplemented with 0.2% glucose. Next, each test agent was diluted in 0.01% acetic acid and 0.2% BSA. Individual wells of sterile, polystyrene, 96-well-flat bottom TCPs were filled with 90 μL of *P. aeruginosa* (1 × 10^6^ CFU/mL) cells, after which 10 μL of test agent was added, with the test agent concentration in the range of 512 to 1 μM. For control, no test agents were added in the wells. The cells in performed biofilms were then incubated for 24 h at 37 °C and the wells were carefully washed with PBS to eliminate free-floating bacteria. The formation of bottom biofilm was fixed with 100 μL of 100% methanol and then incubated for 15 min. After the removal of methanol residue, biofilms in the plate were stained with 0.1% crystal violet for 30 min. Excess stain was thoroughly rinsed off with distilled water and then plates were left to dry. After drying, 95% ethanol was added to each well and OD_590_ of stained biofilm was measured with Versa-Max microplate Elisa Reader (Molecular Devices, Sunnyvale, CA, USA). These optical density values were considered as a measure of bacteria adhering to the surface and forming biofilms. The percentage of biofilm inhibition was calculated using the following equation: [1 − (OD_590_ of cells treated with test agent/OD_590_ of non-treated control)] × 100 [48]. MBIC was defined as the lowest concentration that showed 100% inhibition of the formation of the biofilm. Experiments were performed in triplicate and the data was then averaged.

### 3.7. Hemolysis and Cytotoxicity

The hemolytic activity against fresh hRBCs and cytotoxic activity against HaCaT cell (human keratinocyte) and RAW264.7 (macrophage) cells were examined using a previously described method [21,68].

### 3.8. Trp Fluorescence Assay

Large unilamellar vesicles (LUVs) were prepared using the freeze-thaw method, respectively [74]. Briefly, dry lipid films were resuspended in 1–2 mL of appropriate buffer by vortexing. LUVs were prepared through eight freeze-thaw cycles under liquid nitrogen and water bath at 50 °C. After preparation of vesicles, suspensions were then extruded 13 times through 0.2 μM polycarbonate membranes, and lipid concentrations were determined by a standard phosphate assay [75]. 

To characterize the Trp environment of the peptides, we used fluorescence spectroscopy to examine the binding of the peptides to lipid bilayers. Fluorescence emission spectra of Trp residue in peptides were monitored in the presence of PC, PC:CH (2:1, w/w) and PC:SM (2:1, w/w) as previously described [76]. East peptide was added to 1 mL of 200 μM liposomes, and the peptide:liposome mixture (a molar ratio of 1:100) was allowed to interact at 25 °C for 10 min. The Trp fluorescence measurements were taken using a spectrofluorometer (Perkin-Elmer LS55, Mid Glamorgan, UK) at an excitation wavelength of 280 nm and an emission wavelength ranging from 300 to 400 nm. 

### 3.9. Calcein Leakage

Calcein leakage assay was performed to investigate interactions between AMPs and model liposomes [77]. LUVs with entrapped calcein in a suspension containing 100 μM lipid were then incubated for 25 min with various concentrations of melittin or NRC-16 (0.625–10 μΜ). The permeabilizing activity of peptides was assayed by measuring calcein leakage from LUVs with entrapped calcein using a previously described method [68,76,78].

### 3.10. CD Spectroscopy

The CD spectra were recorded on a Jasco 810 spectropolarimeter (Jasco, Tokyo, Japan) equipped with a temperature control unit using a 0.1-cm path-length quartz cell at 25 °C between 190 and 250 nm. The CD spectra were measured for peptide samples (50 μM) that were dissolved in PBS (pH 7.2) containing 1-mM PC, 1-mM PC:CH (2:1, w/w) vesicles or 1-mM PC:SM (2:1, w/w) vesicles. CD data represent the average value from three separate recordings, with four scans per sample. All CD spectra shown have had the corresponding peptide-free solvent baselines subtracted. The results are expressed in terms of molar residue CD.

## 4. Conclusions

In summary, peptide NRC-16 displayed antimicrobial activity against a wide range of Gram-negative bacteria, Gram-positive bacteria and fungal strains, including clinically isolated bacterial strains such as *P. aeruginosa* and *S. aureus*. This peptide clearly inhibited biofilm formation from *P. aeruginosa*. Furthermore, unlike melittin, NRC-16 peptide showed no cytotoxicity due to its non-interaction with eukaryotic membranes. NRC-16 may thus prove beneficial in human medicine, and be possibly used for the inhibition of bacterial biofilm infection at wound sites.
